# Fownes’ Manual of Chemistry

**Published:** 1878-11

**Authors:** 


*Fownes' Manual of Chemistry, Theoretical and Practical.* Revised and corrected by Henry Watts, B. A., F. R. S., Editor of the Journal of the Chemical Society, etc. New American, from the Twelfth English Edition. Edited by Robert Bridges, M. D., Professor of Chemistry in the Philadelphia College of Pharmacy. With illustrations. Philadelphia: H. C. Lea. Royal 12mo. pp. 1027.

Nearly thirty years have now elapsed since the death of the original author of this work. Regularly renewed improvement and enlargement has however long maintained the standard excellence of the volume. Mr. Watts, the English reviser, has given many evidences of his distinguished ability in the work he has done upon this edition. An increase of one hundred and seventy pages has been made over the number of the former edition, and this concerns principally the department of organic chemistry. The mechanical execution of the volume is most excellent, and gives it a very inviting appearance. The headings of the sections have been set in heavy-faced type with much advantage. The part relating to atomicity and the laws of chemical combinations have been carefully revised and improved, and these subjects are now presented with great clearness and fulness. There appears here an adaptation to the student's standpoint that is not often secured by writers. The science of chemistry has increased its borders to a wonderful extent, and with this the advantage and necessity has become greater of relying upon such a book as this, whose character for comprehensiveness, systematic treatment and ability is unsurpassed.

